# Increase in Metabolic Syndrome-Related Hospitalizations in Relation to Environmental Sources of Persistent Organic Pollutants

**DOI:** 10.3390/ijerph8030762

**Published:** 2011-03-04

**Authors:** Alexander V. Sergeev, David O. Carpenter

**Affiliations:** 1 Department of Social and Public Health, Ohio University, Grover Center W343, Athens, OH 45701, USA; 2 Institute for Health and the Environment, University at Albany, Five University Place, A217, Rensselaer, NY 12144, USA; E-Mail: carpent@uamail.albany.edu

**Keywords:** persistent organic pollutants, metabolic syndrome, hazardous waste sites, residential proximity

## Abstract

Evidence from cell studies indicates that persistent organic pollutants (POP) can induce insulin resistance, an essential component of the metabolic syndrome (MetS). We hypothesized that residential proximity to environmental sources of POP would be associated with the MetS in the population. The present study examined the association between residency in a zip code containing or abutting environmental sources of POP and MetS-related hospitalization rates. Hospitalization data were obtained from the New York Statewide Planning and Research Cooperative System. Relative risks (RR) were calculated as hospitalization rate ratios. Adjusted RR and their 95% confidence intervals (CI) were estimated by multivariable Poisson regression. A higher proportion of African Americans resided in POP zip codes compared to Caucasians (25.9% and 24.3%, respectively, p < 0.01). Residence in POP zip codes was associated with a statistically significant 39.2% increase in MetS-related hospitalization rates, adjusted for race, gender, and age (adjusted RR = 1.392, 95% CI: 1.032–1.879, p = 0.030). Increase in age was independently associated with higher MetS-related hospitalization rates (p for trend < 0.001). Our findings contribute to the body of evidence supporting the hypothesis of POP constituting an environmental risk factor for the MetS. Further studies investigating exposure to POP and insulin resistance are warranted.

## 1. Introduction

The metabolic syndrome (MetS) constitutes a growing problem of a serious public health and health care concern not only because of its increase in the population in recent years, but also because it’s a major risk factor for cardiovascular diseases, which is the leading cause of death in the United States and worldwide [1,2]. Age-adjusted prevalence of the MetS in the U.S. adult population has grown from below 24% in 1988–1994 to 34% in 2003–2006 [3,4]. The MetS is a clustering of metabolism-related disorders and conditions that may share common pathophysiological mechanisms: including glucose intolerance/insulin resistance, abdominal obesity, dyslipidemia, and high blood pressure—hypertension (HTN) [5,6]. Insulin resistance and obesity play the central role in the pathophysiology of the MetS. A formalized clinical definition of the MetS includes having any three of the following five criteria: waist circumference over 40 inches (men) or over 35 inches (women); fasting glucose 100 or higher mg/dL or currently on medication for an elevated glucose level; triglycerides 150 or higher mg/dL or currently on medication for hypertriglyceridemia; high-density lipoprotein (HDL) cholesterol below 40 mg/dL (men) or below 50 mg/dL (women) or currently on medication for low HDL cholesterol level; systolic blood pressure over 130 mm Hg or diastolic blood pressure over 85 mm Hg or currently on medication for HTN [7].

The MetS increases risk of myocardial infarction (heart attack) and stroke; it is also associated with an increased cardiovascular disease mortality and all-cause mortality [8,9].

Persistent organic pollutants (POP) are a group of organic compounds that are highly resistant to chemical, photolytic, and biological degradation. These lipophilic compounds are persistent in the environment and human body. POP include polychlorinated biphenyls (PCB), polychlorinated dibenzo*-p-*dioxins [e.g., 2,3,7,8-tetrachlorodibenzo*-p-*dioxin (TCDD)], polychlorinated dibenzofurans, and persistent pesticides, such as dichlorodiphenyltrichloroethane (DDT) [10]. PCBs were manufactured in the U.S. from 1929 through 1976 for a variety of industrial purposes as determined by the compounds’ low electrical and thermal conductivity. PCBs were used in dielectric fluids in capacitors and transformers; they have also been used in plasticizers, adhesives, paints, pesticide extenders, hydraulic fluids, flame retardants and dyes for carbonless copy paper [11,12]. Inadequate manufacturing control, flaws in waste management, old electrical equipment disposal, and garbage incineration are among sources of environmental contamination with POP [13]. POP are known carcinogens and endocrine disruptors; they have neurotoxicity and immunotoxicity properties and can cause birth defects [12,14].

Recent cell studies reported that POP can interfere with insulin signaling pathway and induce insulin resistance [15,16]. Results from a limited number of studies are indicative of serum levels of POP being associated with MetS prevalence [17,18]. This prompted us to investigate MetS-associated hospitalizations in relation to environmental contamination with POP. We hypothesized that residency in areas containing environmental sources of POP will be associated with an increase in MetS-associated hospitalization rates.

## 2. Methods

### 2.1. Study Population

We conducted a population-based semi-ecological study of MetS-related hospitalizations in relation to presumed environmental exposure to POP, based on zip codes of residence. Semi-ecological designs are used widely in environmental exposure studies. They utilize exposure status measurement on a group level combined with confounder measurement (such as patients’ gender, age, and race) on an individual level [19,20]. Patients residing in zip codes containing or abutting POP-contaminated waste sites were considered environmentally exposed to POP. Hospitalization data were obtained from the New York State (NYS) Statewide Planning and Research Cooperative System (SPARCS) database maintained by the NYS Department of Health. SPARCS is an administrative database; it contains formalized and coded data derived from hospital charts. All state-regulated hospitals in NYS report each patient’s medical (the principal diagnosis and up to 14 secondary diagnoses) and demographical (such as patient’s age, gender, and race) information to the NYS Department of Health. The few hospitals that are exempt from this systematic reporting are federal hospitals, including the ones operated by the U.S. Department of Veterans Affairs.

Both the principal and secondary diagnoses are coded according to the International Classification of Diseases, the Ninth Revision, Clinical Modification (ICD-9-CM). We used data for a three-year period of 2002–2004 for NYS except for New York City. New York City maintains its own hospitalization database and its population structure is different from the rest of the NYS. MetS-related hospitalizations were identified as hospitalizations containing the ICD-9-CM diagnosis code 277.7 as either the principal or a secondary diagnosis in the hospital discharge record. This ICD-9-CM code for the MetS was approved by the National Center for Health Statistics in 2001 [21].

Patients’ environmental exposure status was designated as “POP” or “non-POP” based on their residence in zip codes containing or abutting hazardous waste sites known to contain dioxins/furan, PCBs or persistent pesticides; details of these classifications were described elsewhere [22]. Zip codes’ “POP” status was determined based on the data on 818 hazardous waste sites identified by the New York State Department of Environmental Conservation as “State Superfund” sites; additionally, the zip codes abutting POP-contaminated portions of the Hudson River and the six Areas of Concern—polluted bodies of water as defined by the U.S.-Canadian Joint Commission—were also classified as “POP” zip codes [23]. Lower socio-economic status is often associated with a decreased access to health care, as many of these patients cannot afford to seek medical help and hospitalization. Consequently inclusion of persons of lower socio-economic status often distorts results due to the misbalanced underrepresentation of the disadvantaged population among hospitalized patients, especially in cases of a non-acute or immediately life-threatening illness. Restriction of the population studied by income is a valid and commonly used method to control for socio-economic status [24,25]. Thus in this study we restricted our analysis to those zip codes only in the three upper quartiles of median household income. After the income restriction was applied, there were 117 “POP” zip codes in the study and 584 “non-POP” zip codes. Of the “non-POP” zip codes, 133 zip codes contained or abutted sources of environmental contaminants other than POP, and 451 zip codes contained or abutted no known hazardous waste sites.

Data on race, gender, age, and income by zip code were obtained from Claritas, Inc., derived from the US Census. Because patients of the two largest racial groups—Caucasians and African Americans—comprised more than 98% of the residential NYS population exclusive of New York City and accounted for more than 98% of the MetS-associated hospitalizations, other racial groups were not included for the purpose of minimizing extra variability and preserving the parsimony of the model.

After the above restrictions were applied, there were a total of 408 MetS-associated hospitalizations among 25-years and older adults during 18,113,583 person-years, over a three-year period of 2002–2004.

### 2.2. Statistical Analysis

The primary outcome variable was the MetS-associated hospitalization rate. Hospitalization rates (per 100,000 person-years, over a three-year period) were calculated as a number of MetS-associated hospitalizations divided by the population residing in a given zip code multiplied by 100,000.

Relative risks (RR) of MetS-associated hospitalization in relation to POP exposure status were calculated as hospitalization rate ratios. The multivariable Poisson regression was utilized to obtain RR of MetS-associated hospitalization, with respective 95% confidence intervals (95% CI). Multivariable adjusted analysis was used to control for confounding by age, race, and gender by including the respective variables into the multivariable model.

All statistical analyses were performed with SAS software, version 9.2 (SAS Institute Inc., Cary, NC). The PROC GENMOD procedure was used to conduct the multivariable Poisson regression. To adjust for overdispersion, a scaling factor was used; the scaled Pearson chi-square was equal 1. A conventional p < 0.05 value (type I error alpha = 0.05) was used for all analyses.

## 3. Results

### 3.1. Patient Characteristics

The mean ± SD age of the patients was 58.9 ± 14.4 years. Almost 55% of hospitalized patients were women (224 cases) and about 45% were men (184 cases). Of the total of 408 MetS-associated hospitalizations over a three-year period, 16 (4%) were African American and 392 (96%) Caucasian, which reflects the fact that African Americans constitute 6% and Caucasians 94% of the study population. Table 1 shows the distribution of the demographic characteristics of the study population in relation to presumed environmental exposure status. While of the total study population, 24.4% resided in zip codes containing or abutting environmental sources of POP, race-stratified analysis indicated that this proportion was statistically significantly higher among African Americans than Caucasians (25.9% and 24.3% residing in POP zip codes, respectively; p < 0.01).

Both unadjusted (not controlled for confounding) and adjusted (controlled for confounding) analyses were conducted. Unadjusted analyses provided preliminary information on the distribution of MetS-associated hospitalizations across different race, age, and gender groups. Adjusted analyses were conducted afterwards for the purpose of investigating whether an association between residence in a POP zip code and MetS-associated hospitalizations exists.

### 3.2. Unadjusted Analysis

While unadjusted MetS-associated hospitalization rates were lower in African Americans than in Caucasians, the difference was not statistically significant (unadjusted rates per 100,000 person-years: 1.48 and 2.30, respectively; unadjusted RR = 0.643, 95% CI: 0.390–1.059; p = 0.083) (Table 2). Unadjusted analysis of gender differences in MetS-associated hospitalizations (Table 2) indicated that males have a slightly lower unadjusted rate (2.15 per 100,000 person-years) than females (2.34 per 100,000 person-years), with the difference not being statistically significant (p = 0.398).

Unadjusted analysis of MetS-associated hospitalizations in relation to age indicated a statistically significant increase of hospitalization rates with age (Table 2). Different patterns in hospitalization rates were observed for ages up to 44 years and 45 years and older. MetS-associated hospitalization rates in the former groups (0.31 to 0.79 per 100,000 person-years) were lower than the average hospitalization rate (2.18 per 100,000 person-years), while in the latter age groups they exceeded the average and ranged from 2.84 to 4.21 per 100,000 person years (Table 2).

Stratified unadjusted analyses, comparing MetS-associated hospitalization rates in the populations of POP and non-POP zip codes, were conducted separately for race, gender, and age categories (Table 3). Both African Americans and Caucasians residing in POP zip codes had higher hospitalization rates; however, in African Americans the effect of the presumed exposure status was not statistically significant (unadjusted RR = 1.303, 95% CI: 0.268–3.360, p = 0.623), while in Caucasians it was (unadjusted RR = 1.421, 95% CI: 1.137–1.746, p < 0.001). The presumed POP exposure status was also associated with an increase in MetS-related hospitalizations in both men and women; it was statistically significant for women (unadjusted RR = 1.609, 95% CI: 1.204–2.099, p < 0.01), but not for men (unadjusted RR = 1.194, 95% CI: 0.840–1.618, p = 0.280). Analysis of hospitalization rates by age indicated that in all but the youngest (ages 25–34 years) of the six age categories, the presumed exposure to POP was associated with an increase in MetS-related hospitalizations, although not statistically significant.

**Figure 1.**
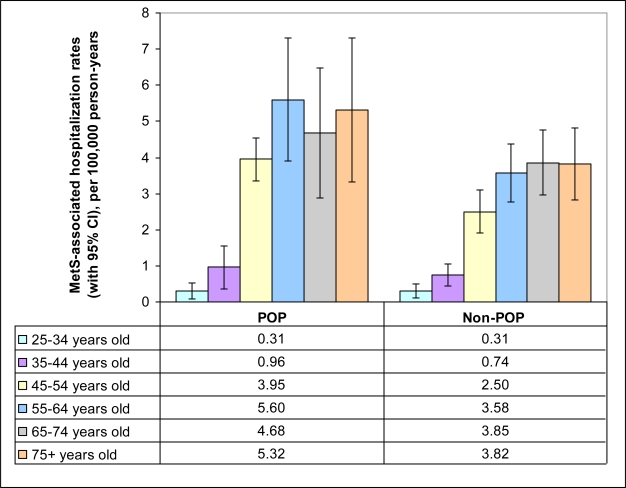
Unadjusted MetS-associated hospitalization rates (per 100,000 person-years) in relation to POP status, by age.

The two different patterns of MetS-associated hospitalizations by age described above for the entire study population were also found in a stratified analysis. Hospitalization rates in the two youngest categories were below the respective POP and non-POP average hospitalization rates (POP average: 2.89 per 100,000 person-years; non-POP average: 2.05 per 100,000 person-years), while for the ages of 45 years and older they were above average (Figure 1) for residents of both POP and non-POP zip codes.

### 3.3. Multivariable Adjusted Analysis

While unadjusted analysis provides useful information on the distribution of hospitalization rates across different population groups, it cannot be used for making conclusions on whether hospitalization rates are associated with exposure, because the results are not controlled for confounding. To investigate whether residence in POP zip codes is associated with an increase in MetS-associated hospitalization rates after controlling (adjusting) for race, gender and age, we conducted a multivariable adjusted analysis, using a multivariable Poisson regression model.

Results of the multivariable Poisson regression analysis are presented in Table 4. After adjusting for race, gender, and age, residence in a POP zip code was associated with a statistically significant 39.2% increase in MetS-related hospitalization rates (adjusted RR = 1.392, 95% CI: 1.032–1.879, p = 0.030). After controlling for the POP exposure status, gender, and age, race was no longer associated with increased hospitalization rates (p = 0.609). After controlling for POP exposure status, race, and age, gender also was not associated with MetS-related hospitalization rates. Age, however, remained associated with an increase in hospitalization rates in the multivariable adjusted analysis (p for trend < 0.001).

## 4. Discussion

In this study we found that residence in zip codes containing or abutting environmental sources of POP is associated with a statistically significant 39.2% increase in MetS-related hospitalizations compared to residents of non-POP zip codes. To our knowledge, this is the first study investigating the association between the presumed environmental exposure to POP based on residence near to a POP hazardous waste site and MetS employing hospitalization rate as the outcome of interest.

We chose to focus on this outcome for two reasons. Firstly, while an increased MetS-related hospitalization rate does not prove a causal effect of POP, it is reasonable to expect that the hypothesized association between POP and MetS does exist. Our previous studies using hospitalization in relation to residence near to POP sites have reported elevated risk of diabetes [26], hypertension [22], and cardiovascular [27] and cerebrovascular disease [28,29]. In addition, studies in which PCBs and pesticides were measured in serum have demonstrated statistically significant relations between exposure and diabetes [30,31], obesity [32], hyperlipidemia [33] and hypertension [34], each one component of MetS. Secondly, the concept of the environmental burden of disease is important from the public health and health care perspectives [35,36], and hospital utilization is an essential component of this concept.

Our results are consistent with findings from other population-based studies of exposure to POP and MetS, or MetS components, that were conduced using different approaches. Chang *et al.* (2011) investigated insulin resistance in non-diabetic adults residing near a deserted pentachlorophenol factory that generated polychlorinated dibenzo*-p-*dioxins and furans as byproducts. They found a statistically significant positive association between serum dioxin levels and homeostasis model assessment—insulin resistance (HOMA-IR; higher values indicative of insulin resistance), fasting glucose, systolic and diastolic blood pressure, waist circumference, and HDL cholesterol level [37].

Cranmer *et al.* (2000) studied association between environmental residential exposure to TCDD and insulin levels in 69 subjects with normal glucose tolerance test who lived within 25 miles of a Superfund site in Arkansas [38]. The source of TCDD contamination was a plant where the TCDD-containing component of Agent Orange was manufactured and from which TCDD spread into the environment due to lack of adequate production control and waste disposal. High blood TCDD levels were found to be associated with higher levels of both fasting insulin and insulin at 30, 60, and 120 minutes after oral glucose load.

Using the National Health and Nutrition Examination Survey data, Lee *et al.* (2007) investigated cross-sectional associations between POP and MetS among 721 adults, of whom 175 individuals had MetS [17]. They found that serum concentrations of organochlorine pesticides and dioxin-like polychlorinated biphenyls (PCBs) were positively associated with the prevalence of the MetS. The dose-response relationship between non-dioxin-like PCBs and the MetS was inverted-U-shaped, with the association being the strongest for the two middle quartiles of serum POP levels. Serum levels of other POP investigated in that study (polychlorinated dibenzofurans and polychlorinated dibenzo*-p-*dioxins) were found to be associated with only one of the MetS components—HTN.

In a study of 160 MetS patients among 1,374 Japanese residents non-occupationally exposed to POP, Uemura *et al.* (2009) found that body burden levels of polychlorinated dibenzofurans, polychlorinated dibenzo*-p-*dioxins, and dioxin-like PCBs were statistically significantly associated with the prevalence of the MetS [18].

While the exact pathogenic mechanisms involved in the association between POP and the MetS have not been established with certainty, results from a number of studies indicate that insulin resistance and changes in gene regulation caused by POP play the major role. POP can increase secretion of tumor necrosis factor alpha (TNF-α), which plays an essential role in development of insulin resistance. The insulin resistance-inducing effect of TNF-α is established [39–41]. 2,3,7,8-Tetrachlorodibenzo*-p-*dioxin (TCDD) has been shown to stimulate TNF-α and inhibit glucose transport in adipose cells (adipocytes) [15,42]. TCDD interferes with the insulin signaling pathway by decreasing the expression levels of insulin receptors and glucose transporter GLUT-4 that results in inhibiting insulin-stimulated glucose uptake [16]. The underlying mechanism of the insulin resistance-inducing effect of TCDD is stimulation of TNF-α production [16]. TNF-α substantially decreases GLUT-4 [43]. TNF-α activates nuclear factor kappa B (NF-κB)—an obligatory mediator of the changes in gene expression induced by TNF-α, including GLUT-4. This pathway leads to development of insulin resistance [44].

The role of the TNF-α → NF-κB → GLUT4 pathway in POP-induced glucose intolerance was also demonstrated in a study of United States Air Force veterans of the Vietnam War who were involved with spraying the Agent Orange herbicide that was contaminated with TCDD. Fujiyoshi *et al.* (2006) investigated the NF-κB:GLUT4 ratio in the exposed veterans, as compared to the veterans without history of exposure, and found that this ratio is significantly correlated with serum dioxin levels [45]. In another study of the exposed veterans, Michalek *et al.* (1999) found elevated levels of insulin in non-diabetic veterans [46]. High insulin levels would be expected in the MetS.

Although investigating MetS-associated hospitalization in relation to demographic characteristics was not a goal of our study, these results may be worth a brief discussion. Prevalence of the MetS is known to increase with age [4,47]. Our findings are consistent with this knowledge, in that MetS-associated hospitalization rates increased with age (p for trend < 0.001). While MetS prevalence is higher in men than in women and in Caucasians than in African Americans, gender and racial differences in the MetS components vary substantially [4,48]. While hypertriglyceridemia, HTN, and high fasting glucose are more prevalent in men, abdominal obesity and low HDL cholesterol are more prevalent in women [4]. Abdominal obesity, hypertriglyceridemia, low HDL cholesterol, and high fasting glucose are more prevalent in Caucasians, but HTN is more prevalent in African Americans [4]. In our study, racial and gender differences in MetS-associated hospitalization (higher rates in Caucasians than in African Americans and in men than in women) were not statistically significant. Probably, racial and gender differences appearing in MetS prevalence became smoothed in MetS-associated hospitalization rates because diseases and conditions that do not constitute an immediate threat to patient’s life or risk of permanent and severe disability if not treated promptly may be underrepresented in hospital settings.

Our study is not free from limitations. Exposure status information was available only on a zip code-level (group level), which is a very crude measure of exposure. Group-level measurements are a common limitation intrinsic to ecological and semi-ecological studies. Therefore, the results of such studies cannot be extrapolated to individual level because of ecological fallacy problem [49,50]. It should be noted, however, that ecological design remains valuable for studies investigating population-level processes and effects, such as an extent of public health impact of social processes and outcomes of public health interventions [49,51]. Only data on hospital discharges from State-regulated hospitals were used, as data from Federal hospitals were not available. We also utilized only the ICD-9 code for MetS, not that for the components of the disease. This may have resulted in an underestimation of the true rate of the MetS.

It can be reasonably argued that crude measurement of exposure might have produced a dilution effect, thus weakening the measure of association (underestimation of RR) due to non-differential misclassification. That means that if exposure status was measured more accurately, the true RR obtained in such a study would have been even higher, indicating stronger association between exposure and outcome. Since exposure and outcome status were estimated independently from each other, using independent sources of data, differential misclassification is unlikely in our study.

## 5. Conclusions

In conclusion, our findings contribute to the body of evidence supporting the hypothesis of POP constituting an environmental risk factor for the MetS. Further studies of POP exposure and the MetS with individual level measurements of exposure and outcome, including measures of insulin resistance, are warranted. Given the involuntary nature of environmental residential exposure to POP that may affect a substantial portion of population, it is imperative to develop and implement respective public health and environmental health policy interventions aimed at reducing and eliminating POP exposure.

## Figures and Tables

**Table 1.** Demographic characteristics of the study population by residential proximity to environmental sources of POP (2002–2004), person-years (%).

**Population groups**	**POP**	**Non-POP**	**Total**
*Race:*			
African Americans	279,804 (25.9)	802,119 (74.1)	1,081,923 (100.0)
Caucasians	4,146,579 (24.3)	12,885,081 (75.7)	17,031,660 (100.0)

*Age:*			
25–34 years	977,889 (25.1)	2,913,609 (74.9)	3,891,498 (100.0)
35–44 years	1,046,424 (24.3)	3,262,539 (75.7)	4,308,963 (100.0)
45–54 years	809,217 (22.9)	2,717,067 (77.1)	3,526,284 (100.0)
55–64 years	607,584 (23.7)	1,954,566 (76.3)	2,562,150 (100.0)
65–74 years	533,748 (25.5)	1,557,114 (74.5)	2,090,862 (100.0)
75+ years	451,521 (26.0)	1,282,305 (74.0)	1,733,826 (100.0)

*Gender:*			
males	2,077,314 (24.3)	6,470,253 (75.7)	8,547,567 (100.0)
females	2,349,069 (24.6)	7,216,947 (75.4)	9,566,016 (100.0)

*Total study population*	4,426,383 (24.4)	13,687,200 (75.6)	18,113,583 (100.0)

**Table 2.** Unadjusted hospital admission rates (per 100,000 person-years) and unadjusted relative risks of MetS-associated hospital admission, by race, gender, and age.

**Population characteristics**	**Unadjusted MetS-associated hospital admission rates (95% CI), per 100,000 person-years**	**Unadjusted RR (95% CI)**	**p value**
*Race:*			
Caucasians (reference)	2.30 (2.07–2.53)	1.0	
African Americans	1.48 (0.75–2.20)	0.643 (0.390–1.059)	0.083

*Gender:*			
females (reference)	2.34 (2.03–2.65)	1.0	
males	2.15 (1.84–2.46)	0.919 (0.755–1.116)	0.398

*Age:*			
25–34 years (reference)	0.31 (0.13–0.48)	1.0	<0.01*
35–44 years	0.79 (0.52–1.05)	2.559 (1.325–4.942)	
45–54 years	2.84 (2.28–3.39)	9.197 (5.053–16.737)	
55–64 years	4.06 (3.28–4.84)	13.164 (7.242–23.927)	
65–74 years	4.07 (3.20–4.93)	13.184 (7.204–24.129)	
75+ years	4.21 (3.24–5.18)	13.654 (7.415–25.143)	

*Income (quartiles of median household income):*			
quartile 1 (reference)	4.61 (3.76–5.47)	1.0	<0.01*
quartile 2	2.91 (2.34–3.49)	0.632 (0.480–0.827)	
quartile 3	2.90 (2.47–3.33)	0.629 (0.499–0.804)	
quartile 4	1.54 (1.28–1.80)	0.333 (0.259–0.430)	

* p value for trend, Wald statistic.

**Table 3.** Unadjusted MetS-associated hospital admission rates (per 100,000 person-years) and unadjusted relative risks of hospital admission in relation to residential proximity to environmental sources of POP.

**Population characteristics**	**Unadjusted MetS-associated hospital admission rates (95% CI), per 100,000 person-years**	**Unadjusted RR (95% CI)**	**p value**
*Race:*			
African Americans			
non-POP (reference)	1.37 (0.56–2.18)	1.0	
POP	1.79 (0.22–3.35)	1.303 (0.268–3.360)	0.623
Caucasians			
non-POP (reference)	2.09 (1.84–2.34)	1.0	
POP	2.97 (2.44–3.49)	1.421 (1.137–1.746)	<0.001

*Gender:*			
females			
non-POP (reference)	2.04 (1.71–2.37)	1.0	
POP	3.28 (2.55–4.10)	1.609 (1.204–2.099)	<0.01
males			
non-POP (reference)	2.06 (1.71–2.40)	1.0	
POP	2.46 (1.78–3.13)	1.194 (0.840–1.618)	0.280

*Age:*			
25–34 years			
non-POP (reference)	0.31 (0.11–0.51)	1.0	
POP	0.31 (0.06–0.65)	0.993 (0.015–2.920)	0.992
35–44 years			
non-POP (reference)	0.74 (0.44–1.03)	1.0	
POP	0.96 (0.36–1.55)	1.299 (0.512–2.523)	0.486
45–54 years			
non-POP (reference)	2.50 (1.91–3.10)	1.0	
POP	3.95 (2.58–5.32)	1.580 (0.995–2.347)	0.186
55–64 years			
non-POP (reference)	3.58 (2.74–4.42)	1.0	
POP	5.60 (3.71–7.48)	1.563 (0.998–2.302)	0.186
65–74 years			
non-POP (reference)	3.85 (2.88–4.83)	1.0	
POP	4.68 (2.85–6.52)	1.216 (0.717–1.873)	0.411
75+ years			
non-POP (reference)	3.82 (2.75–4.89)	1.0	
POP	5.32 (3.19–7.44)	1.391 (0.806–2.200)	0.183

**Table 4.** Multivariable adjusted relative risks of MetS-associated hospitalization in relation to residential proximity to environmental sources of POP and demographic characteristics.

**Parameter**	**β coefficient**	**Standard error**	**Multivariable adjusted RR (95% CI)**	**p value**
POP (compared to non-POP)^1^	0.331	0.153	1.392 (1.032–1.879)	0.030

African Americans (compared to Caucasians)^2^	–0.188	0.366	0.829 (0.404–1.700)	0.609

Males (compared to females)^3^	0.003	0.144	1.003 (0.757–1.328)	0.984

Age (compared to 25–34 years)^4^				<0.001*
35–44 years	0.928	0.481	2.530 (0.985–6.497)	
45–54 years	2.181	0.438	8.857 (3.754–20.897)	
55–64 years	2.523	0.437	12.461 (5.290–29.353)	
65–74 years	2.531	0.442	12.560 (5.278–29.886)	
75+ years	2.568	0.447	13.042 (5.427–31.350)	

1 Adjusted for gender, race, and age;

2 Adjusted for exposure status, gender, and age;

3 Adjusted for exposure status, race, and age;

4 Adjusted for exposure status, gender, and race;

* p value for trend, Wald statistic.
